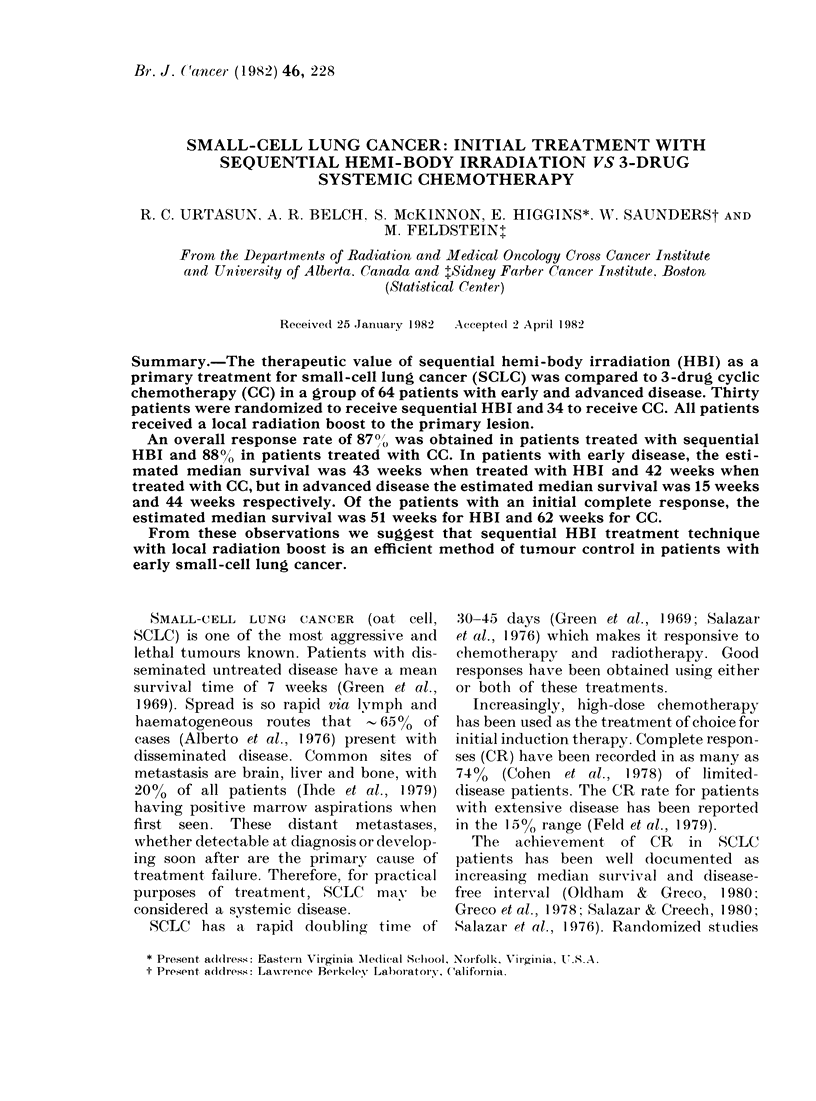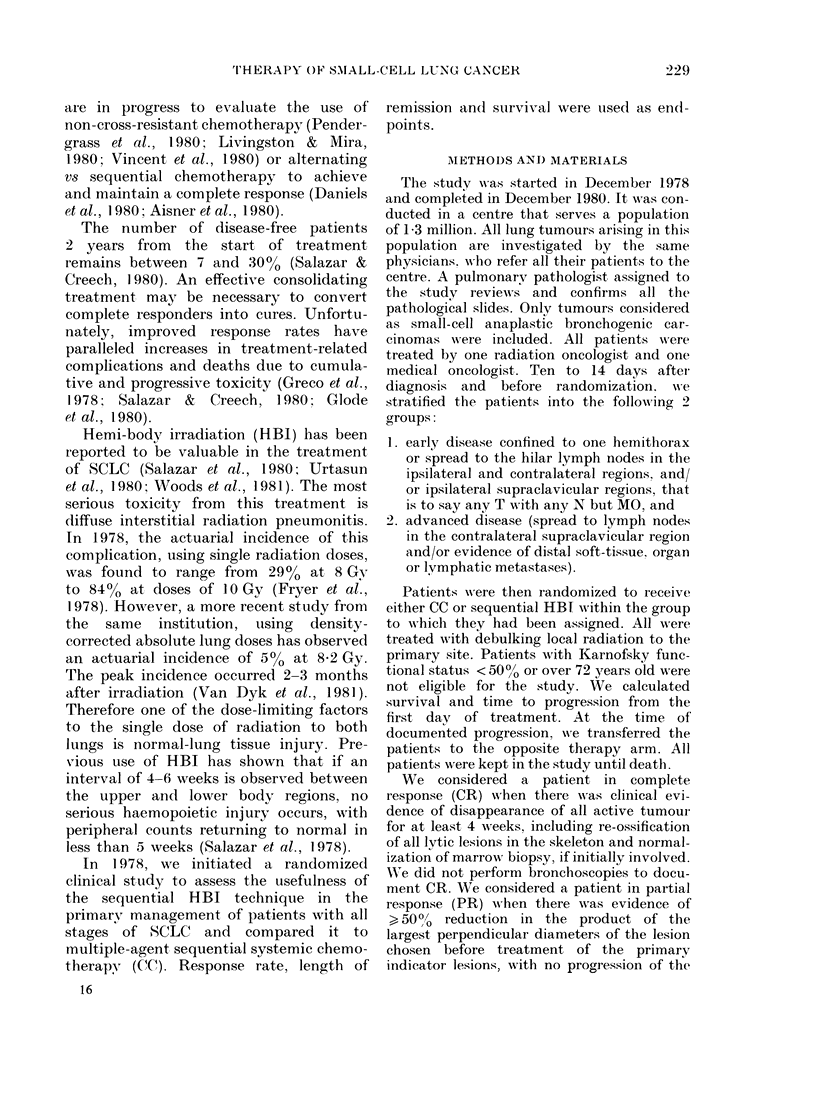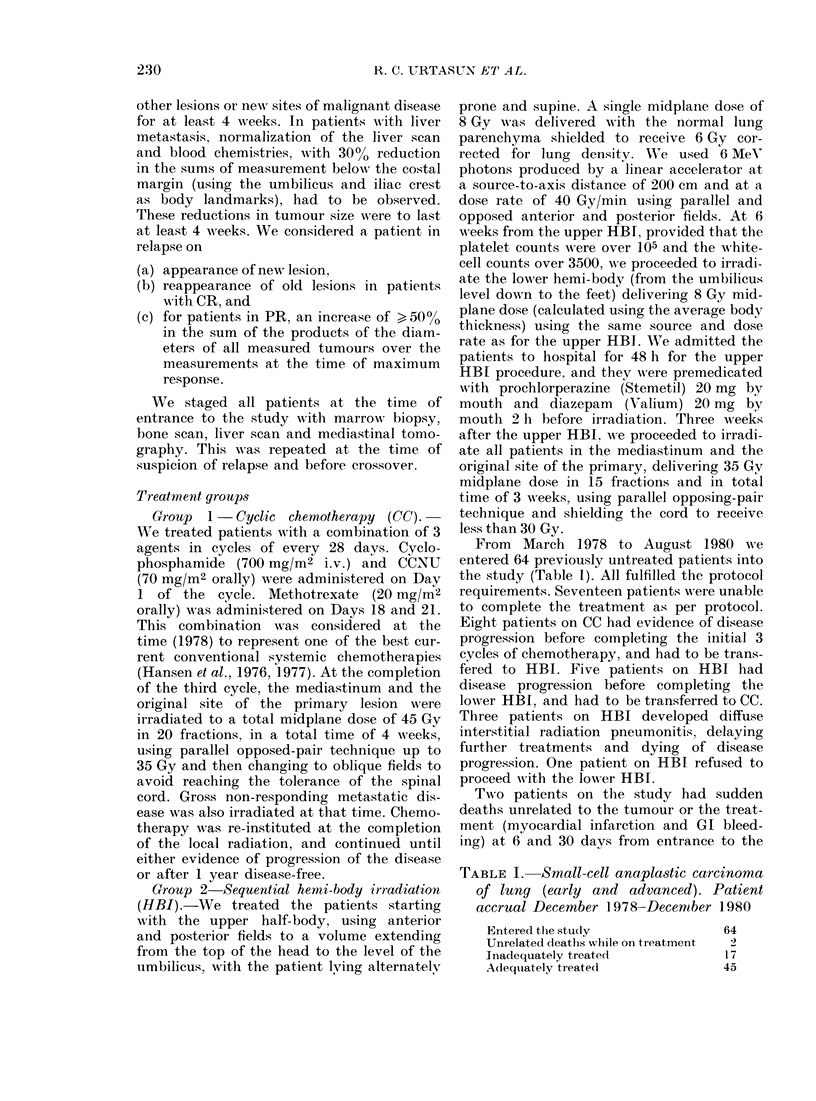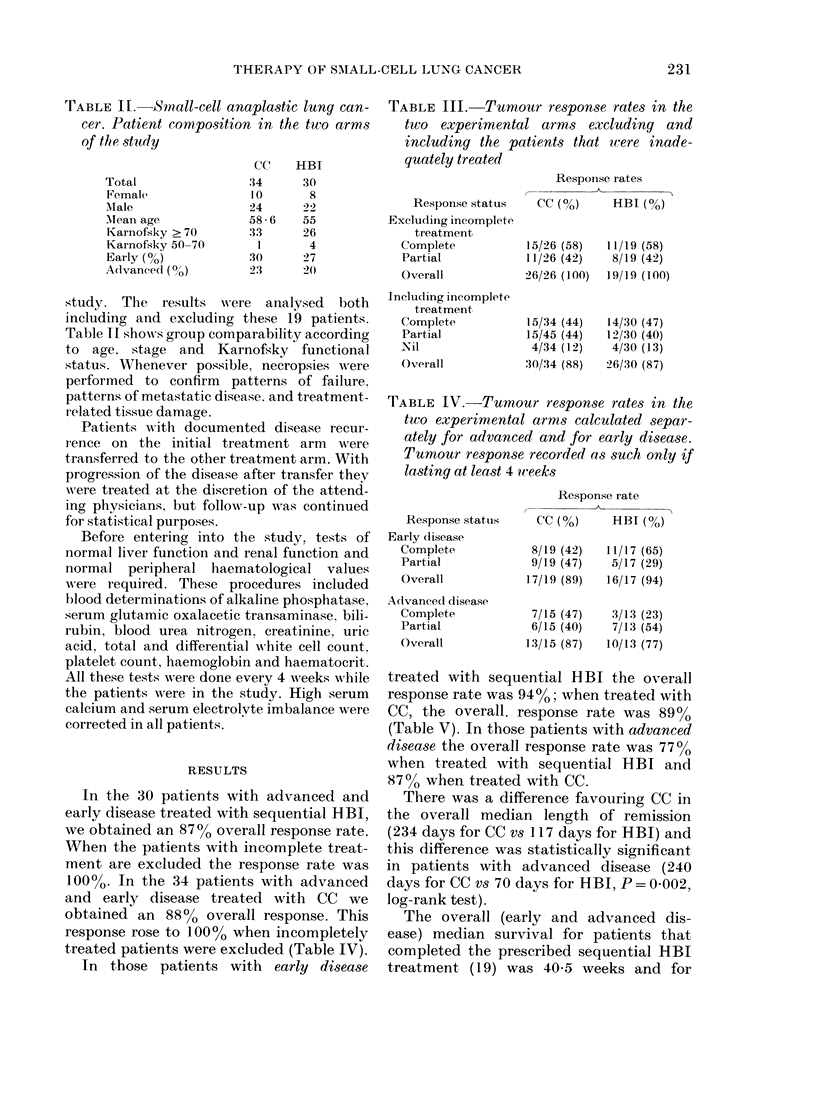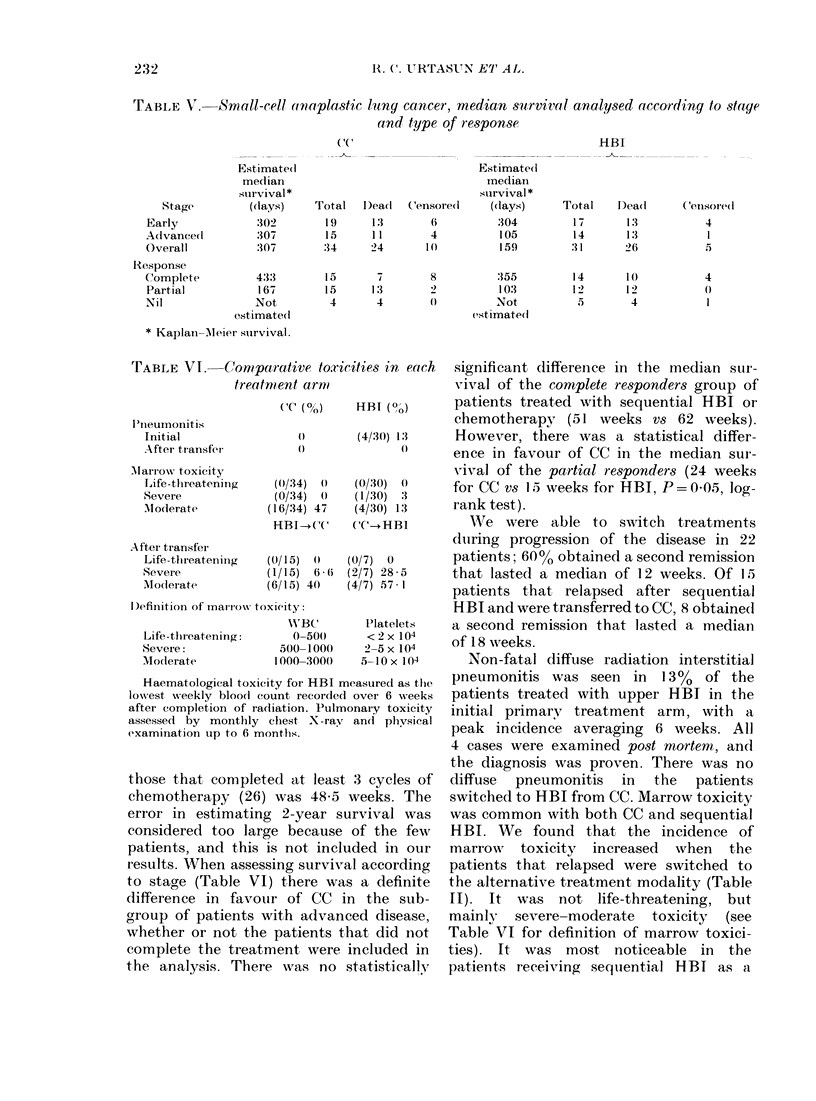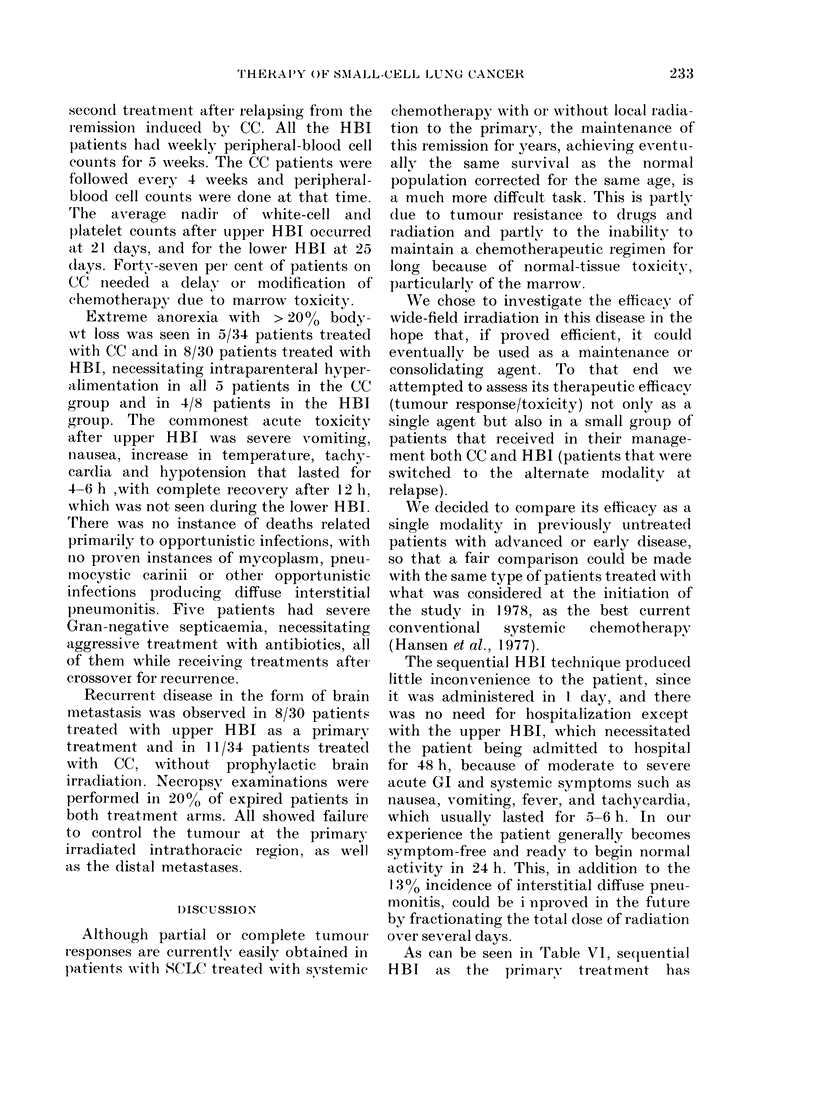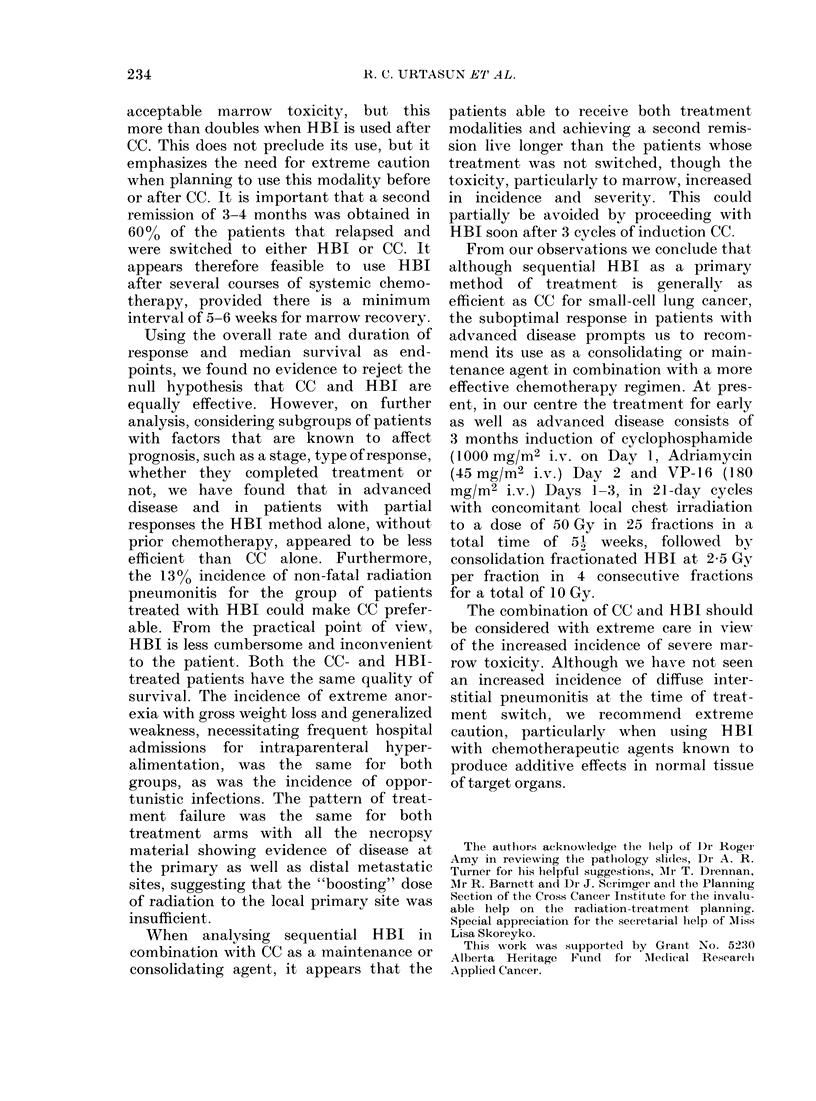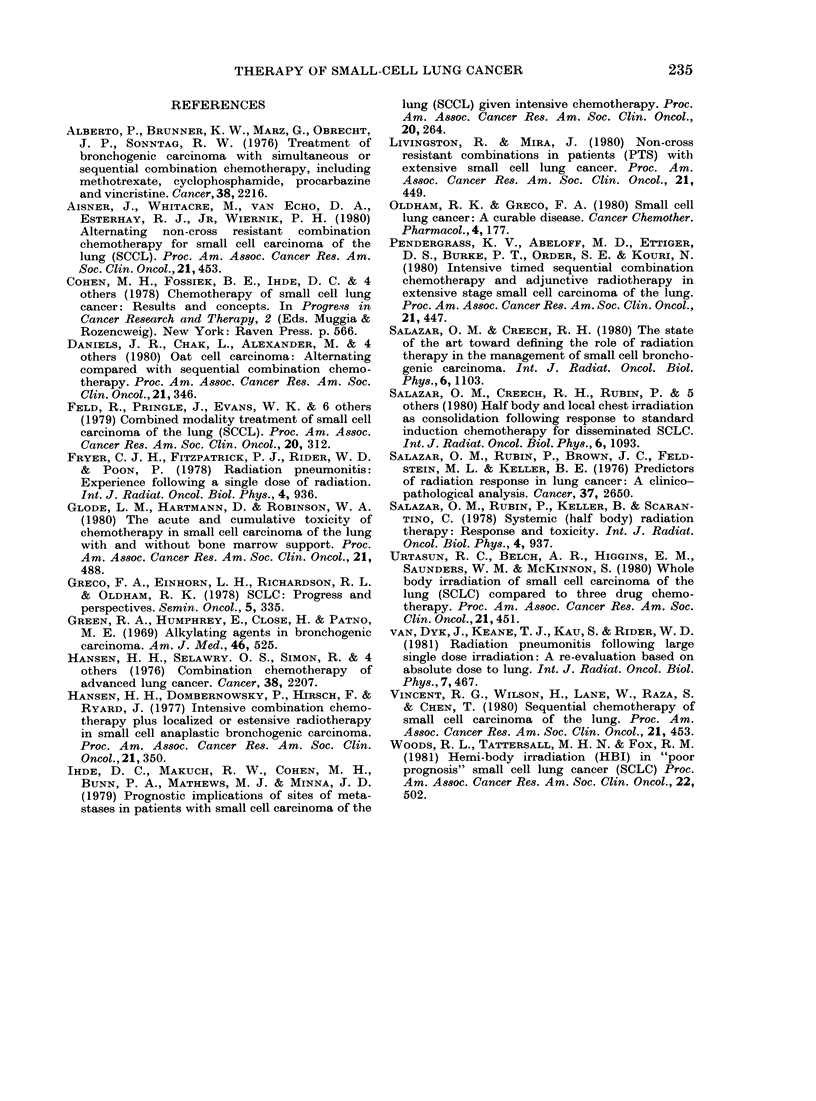# Small-cell lung cancer: initial treatment with sequential hemi-body irradiation VS 3-drug systemic chemotherapy.

**DOI:** 10.1038/bjc.1982.188

**Published:** 1982-08

**Authors:** R. C. Urtasun, A. R. Belch, S. McKinnon, E. Higgins, W. Saunders, M. Feldstein

## Abstract

The therapeutic value of sequential hemi-body irradiation (HBI) as a primary treatment for small-cell lung cancer (SCLC) was compared to 3-drug cyclic chemotherapy (CC) in a group of 64 patients with early and advanced disease. Thirty patients were randomized to receive sequential HBI and 34 to receive CC. All patients received a local radiation boost to the primary lesion. An overall response rate of 87% was obtained in patients treated with sequential HBI and 88% in patients treated with CC. In patients with early disease, the estimated median survival was 43 weeks when treated with HBI and 42 weeks when treated with CC, but in advanced disease the estimated median survival was 15 weeks and 44 weeks respectively. Of the patients with an initial complete response, the estimated median survival was 51 weeks for HBI and 62 weeks for CC. From these observations we suggest that sequential HBI treatment technique with local radiation boost is an efficient method of tumour control in patients with early small-cell lung cancer.


*Br. J. Cancer* (1982) **46**, 228

### SMALL-CELL LUNG CANCER: INITIAL TREATMENT WITH SEQUENTIAL HEMI-BODY IRRADIATION *VS* 3-DRUG SYSTEMIC CHEMOTHERAPY

R. C. URTASUN, A. R. BELCH, S. McKINNON, E. HIGGINS*, W. SAUNDERS† AND M. FELDSTEIN‡

*From the Departments of Radiation and Medical Oncology Cross Cancer Institute and University of Alberta, Canada and ‡Sidney Farber Cancer Institute, Boston (Statistical Center)*

Received 25 January 1982 Accepted 2 April 1982

**Summary.—The therapeutic value of sequential hemi-body irradiation (HBI) as a primary treatment for small-cell lung cancer (SCLC) was compared to 3-drug cyclic chemotherapy (CC) in a group of 64 patients with early and advanced disease. Thirty patients were randomized to receive sequential HBI and 34 to receive CC. All patients received a local radiation boost to the primary lesion.**

**An overall response rate of 87% was obtained in patients treated with sequential HBI and 88% in patients treated with CC. In patients with early disease, the estimated median survival was 43 weeks when treated with HBI and 42 weeks when treated with CC, but in advanced disease the estimated median survival was 15 weeks and 44 weeks respectively. Of the patients with an initial complete response, the estimated median survival was 51 weeks for HBI and 62 weeks for CC.**

**From these observations we suggest that sequential HBI treatment technique with local radiation boost is an efficient method of tumour control in patients with early small-cell lung cancer.**

SMALL-CELL LUNG CANCER (oat cell, SCLC) is one of the most aggressive and lethal tumours known. Patients with disseminated untreated disease have a mean survival time of 7 weeks (Green *et al.*, 1969). Spread is so rapid *via* lymph and haematogeneous routes that ~65% of cases (Alberto *et al.*, 1976) present with disseminated disease. Common sites of metastasis are brain, liver and bone, with 20% of all patients (Ihde *et al.*, 1979) having positive marrow aspirations when first seen. These distant metastases, whether detectable at diagnosis or developing soon after are the primary cause of treatment failure. Therefore, for practical purposes of treatment, SCLC may be considered a systemic disease.

SCLC has a rapid doubling time of 30–45 days (Green *et al.*, 1969; Salazar *et al.*, 1976) which makes it responsive to chemotherapy and radiotherapy. Good responses have been obtained using either or both of these treatments.

Increasingly, high-dose chemotherapy has been used as the treatment of choice for initial induction therapy. Complete responses (CR) have been recorded in as many as 74% (Cohen *et al.*, 1978) of limited-disease patients. The CR rate for patients with extensive disease has been reported in the 15% range (Feld *et al.*, 1979).

The achievement of CR in SCLC patients has been well documented as increasing median survival and disease-free interval (Oldham & Greco, 1980; Greco *et al.*, 1978; Salazar & Creech, 1980; Salazar *et al.*, 1976). Randomized studies

* Present address: Eastern Virginia Medical School, Norfolk, Virginia, U.S.A.

† Present address: Lawrence Berkeley Laboratory, California.

are in progress to evaluate the use of non-cross-resistant chemotherapy (Pendergrass *et al.*, 1980; Livingston & Mira, 1980; Vincent *et al.*, 1980) or alternating *vs* sequential chemotherapy to achieve and maintain a complete response (Daniels *et al.*, 1980; Aisner *et al.*, 1980).

The number of disease-free patients 2 years from the start of treatment remains between 7 and 30% (Salazar & Creech, 1980). An effective consolidating treatment may be necessary to convert complete responders into cures. Unfortunately, improved response rates have paralleled increases in treatment-related complications and deaths due to cumulative and progressive toxicity (Greco *et al.*, 1978; Salazar & Creech, 1980; Glode *et al.*, 1980).

Hemi-body irradiation (HBI) has been reported to be valuable in the treatment of SCLC (Salazar *et al.*, 1980; Urtasun *et al.*, 1980; Woods *et al.*, 1981). The most serious toxicity from this treatment is diffuse interstitial radiation pneumonitis. In 1978, the actuarial incidence of this complication, using single radiation doses, was found to range from 29% at 8 Gy to 84% at doses of 10 Gy (Fryer *et al.*, 1978). However, a more recent study from the same institution, using density-corrected absolute lung doses has observed an actuarial incidence of 5% at 8·2 Gy. The peak incidence occurred 2–3 months after irradiation (Van Dyk *et al.*, 1981). Therefore one of the dose-limiting factors to the single dose of radiation to both lungs is normal-lung tissue injury. Previous use of HBI has shown that if an interval of 4–6 weeks is observed between the upper and lower body regions, no serious haemopoietic injury occurs, with peripheral counts returning to normal in less than 5 weeks (Salazar *et al.*, 1978).

In 1978, we initiated a randomized clinical study to assess the usefulness of the sequential HBI technique in the primary management of patients with all stages of SCLC and compared it to multiple-agent sequential systemic chemotherapy (CC). Response rate, length of remission and survival were used as end-points.

### METHODS AND MATERIALS

The study was started in December 1978 and completed in December 1980. It was conducted in a centre that serves a population of 1·3 million. All lung tumours arising in this population are investigated by the same physicians, who refer all their patients to the centre. A pulmonary pathologist assigned to the study reviews and confirms all the pathological slides. Only tumours considered as small-cell anaplastic bronchogenic carcinomas were included. All patients were treated by one radiation oncologist and one medical oncologist. Ten to 14 days after diagnosis and before randomization, we stratified the patients into the following 2 groups:

1. early disease confined to one hemithorax or spread to the hilar lymph nodes in the ipsilateral and contralateral regions, and/or ipsilateral supraclavicular regions, that is to say any T with any N but MO, and
2. advanced disease (spread to lymph nodes in the contralateral supraclavicular region and/or evidence of distal soft-tissue, organ or lymphatic metastases).

Patients were then randomized to receive either CC or sequential HBI within the group to which they had been assigned. All were treated with debulking local radiation to the primary site. Patients with Karnofsky functional status $<50\%$ or over 72 years old were not eligible for the study. We calculated survival and time to progression from the first day of treatment. At the time of documented progression, we transferred the patients to the opposite therapy arm. All patients were kept in the study until death.

We considered a patient in complete response (CR) when there was clinical evidence of disappearance of all active tumour for at least 4 weeks, including re-ossification of all lytic lesions in the skeleton and normalization of marrow biopsy, if initially involved. We did not perform bronchoscopies to document CR. We considered a patient in partial response (PR) when there was evidence of $\geqslant 50\%$ reduction in the product of the largest perpendicular diameters of the lesion chosen before treatment of the primary indicator lesions, with no progression of the other lesions or new sites of malignant disease for at least 4 weeks. In patients with liver metastasis, normalization of the liver scan and blood chemistries, with 30% reduction in the sums of measurement below the costal margin (using the umbilicus and iliac crest as body landmarks), had to be observed. These reductions in tumour size were to last at least 4 weeks. We considered a patient in relapse on

(a) appearance of new lesion,
(b) reappearance of old lesions in patients with CR, and
(c) for patients in PR, an increase of ≥50% in the sum of the products of the diameters of all measured tumours over the measurements at the time of maximum response.

We staged all patients at the time of entrance to the study with marrow biopsy, bone scan, liver scan and mediastinal tomography. This was repeated at the time of suspicion of relapse and before crossover.

*Treatment groups*

*Group* 1—*Cyclic chemotherapy* (*CC*).—We treated patients with a combination of 3 agents in cycles of every 28 days. Cyclophosphamide (700 mg/m$^2$ i.v.) and CCNU (70 mg/m$^2$ orally) were administered on Day 1 of the cycle. Methotrexate (20 mg/m$^2$ orally) was administered on Days 18 and 21. This combination was considered at the time (1978) to represent one of the best current conventional systemic chemotherapies (Hansen *et al.*, 1976, 1977). At the completion of the third cycle, the mediastinum and the original site of the primary lesion were irradiated to a total midplane dose of 45 Gy in 20 fractions, in a total time of 4 weeks, using parallel opposed-pair technique up to 35 Gy and then changing to oblique fields to avoid reaching the tolerance of the spinal cord. Gross non-responding metastatic disease was also irradiated at that time. Chemotherapy was re-instituted at the completion of the local radiation, and continued until either evidence of progression of the disease or after 1 year disease-free.

*Group* 2—*Sequential hemi-body irradiation* (*HBI*).—We treated the patients starting with the upper half-body, using anterior and posterior fields to a volume extending from the top of the head to the level of the umbilicus, with the patient lying alternately prone and supine. A single midplane dose of 8 Gy was delivered with the normal lung parenchyma shielded to receive 6 Gy corrected for lung density. We used 6 MeV photons produced by a linear accelerator at a source-to-axis distance of 200 cm and at a dose rate of 40 Gy/min using parallel and opposed anterior and posterior fields. At 6 weeks from the upper HBI, provided that the platelet counts were over $10^5$ and the white-cell counts over 3500, we proceeded to irradiate the lower hemi-body (from the umbilicus level down to the feet) delivering 8 Gy midplane dose (calculated using the average body thickness) using the same source and dose rate as for the upper HBI. We admitted the patients to hospital for 48 h for the upper HBI procedure, and they were premedicated with prochlorperazine (Stemetil) 20 mg by mouth and diazepam (Valium) 20 mg by mouth 2 h before irradiation. Three weeks after the upper HBI, we proceeded to irradiate all patients in the mediastinum and the original site of the primary, delivering 35 Gy midplane dose in 15 fractions and in total time of 3 weeks, using parallel opposing-pair technique and shielding the cord to receive less than 30 Gy.

From March 1978 to August 1980 we entered 64 previously untreated patients into the study (Table I). All fulfilled the protocol requirements. Seventeen patients were unable to complete the treatment as per protocol. Eight patients on CC had evidence of disease progression before completing the initial 3 cycles of chemotherapy, and had to be transfered to HBI. Five patients on HBI had disease progression before completing the lower HBI, and had to be transferred to CC. Three patients on HBI developed diffuse interstitial radiation pneumonitis, delaying further treatments and dying of disease progression. One patient on HBI refused to proceed with the lower HBI.

Two patients on the study had sudden deaths unrelated to the tumour or the treatment (myocardial infarction and GI bleeding) at 6 and 30 days from entrance to the

TABLE I.—*Small-cell anaplastic carcinoma of lung* (*early and advanced*). *Patient accrual December* 1978–*December* 1980

| | |
|---|---|
| Entered the study | 64 |
| Unrelated deaths while on treatment | 2 |
| Inadequately treated | 17 |
| Adequately treated | 45 |

TABLE II.—*Small-cell anaplastic lung cancer. Patient composition in the two arms of the study*

| | CC | HBI |
|---|---|---|
| Total | 34 | 30 |
| Female | 10 | 8 |
| Male | 24 | 22 |
| Mean age | 58·6 | 55 |
| Karnofsky ≥70 | 33 | 26 |
| Karnofsky 50–70 | 1 | 4 |
| Early (%) | 30 | 27 |
| Advanced (%) | 23 | 20 |

study. The results were analysed both including and excluding these 19 patients. Table II shows group comparability according to age, stage and Karnofsky functional status. Whenever possible, necropsies were performed to confirm patterns of failure, patterns of metastatic disease, and treatment-related tissue damage.

Patients with documented disease recurrence on the initial treatment arm were transferred to the other treatment arm. With progression of the disease after transfer they were treated at the discretion of the attending physicians, but follow-up was continued for statistical purposes.

Before entering into the study, tests of normal liver function and renal function and normal peripheral haematological values were required. These procedures included blood determinations of alkaline phosphatase, serum glutamic oxalacetic transaminase, bilirubin, blood urea nitrogen, creatinine, uric acid, total and differential white cell count, platelet count, haemoglobin and haematocrit. All these tests were done every 4 weeks while the patients were in the study. High serum calcium and serum electrolyte imbalance were corrected in all patients.

### RESULTS

In the 30 patients with advanced and early disease treated with sequential HBI, we obtained an 87% overall response rate. When the patients with incomplete treatment are excluded the response rate was 100%. In the 34 patients with advanced and early disease treated with CC we obtained an 88% overall response. This response rose to 100% when incompletely treated patients were excluded (Table IV).

In those patients with *early disease*

TABLE III.—*Tumour response rates in the two experimental arms excluding and including the patients that were inadequately treated*

| | Response rates | |
|---|---|---|
| Response status | CC (%) | HBI (%) |
| Excluding incomplete treatment | | |
| Complete | 15/26 (58) | 11/19 (58) |
| Partial | 11/26 (42) | 8/19 (42) |
| Overall | 26/26 (100) | 19/19 (100) |
| Including incomplete treatment | | |
| Complete | 15/34 (44) | 14/30 (47) |
| Partial | 15/45 (44) | 12/30 (40) |
| Nil | 4/34 (12) | 4/30 (13) |
| Overall | 30/34 (88) | 26/30 (87) |

TABLE IV.—*Tumour response rates in the two experimental arms calculated separately for advanced and for early disease. Tumour response recorded as such only if lasting at least* 4 *weeks*

| | Response rate | |
|---|---|---|
| Response status | CC (%) | HBI (%) |
| Early disease | | |
| Complete | 8/19 (42) | 11/17 (65) |
| Partial | 9/19 (47) | 5/17 (29) |
| Overall | 17/19 (89) | 16/17 (94) |
| Advanced disease | | |
| Complete | 7/15 (47) | 3/13 (23) |
| Partial | 6/15 (40) | 7/13 (54) |
| Overall | 13/15 (87) | 10/13 (77) |

treated with sequential HBI the overall response rate was 94%; when treated with CC, the overall. response rate was 89% (Table V). In those patients with *advanced disease* the overall response rate was 77% when treated with sequential HBI and 87% when treated with CC.

There was a difference favouring CC in the overall median length of remission (234 days for CC *vs* 117 days for HBI) and this difference was statistically significant in patients with advanced disease (240 days for CC *vs* 70 days for HBI, $P = 0{\cdot}002$, log-rank test).

The overall (early and advanced disease) median survival for patients that completed the prescribed sequential HBI treatment (19) was 40·5 weeks and for

TABLE V.—*Small-cell anaplastic lung cancer, median survival analysed according to stage and type of response*

| | CC | | | | HBI | | | |
|---|---|---|---|---|---|---|---|---|
| Stage | Estimated median survival* (days) | Total | Dead | Censored | Estimated median survival* (days) | Total | Dead | Censored |
| Early | 302 | 19 | 13 | 6 | 304 | 17 | 13 | 4 |
| Advanced | 307 | 15 | 11 | 4 | 105 | 14 | 13 | 1 |
| Overall | 307 | 34 | 24 | 10 | 159 | 31 | 26 | 5 |
| Response | | | | | | | | |
| Complete | 433 | 15 | 7 | 8 | 355 | 14 | 10 | 4 |
| Partial | 167 | 15 | 13 | 2 | 103 | 12 | 12 | 0 |
| Nil | Not estimated | 4 | 4 | 0 | Not estimated | 5 | 4 | 1 |

* Kaplan–Meier survival.

TABLE VI.—*Comparative toxicities in each treatment arm*

| | CC (%) | HBI (%) |
|---|---|---|
| Pneumonitis | | |
| Initial | 0 | (4/30) 13 |
| After transfer | 0 | 0 |
| Marrow toxicity | | |
| Life-threatening | (0/34) 0 | (0/30) 0 |
| Severe | (0/34) 0 | (1/30) 3 |
| Moderate | (16/34) 47 | (4/30) 13 |
| | HBI→CC | CC→HBI |
| After transfer | | |
| Life-threatening | (0/15) 0 | (0/7) 0 |
| Severe | (1/15) 6·6 | (2/7) 28·5 |
| Moderate | (6/15) 40 | (4/7) 57·1 |

Definition of marrow toxicity:

| | WBC | Platelets |
|---|---|---|
| Life-threatening: | 0–500 | $<2\times10^4$ |
| Severe: | 500–1000 | $2–5\times10^4$ |
| Moderate | 1000–3000 | $5–10\times10^4$ |

Haematological toxicity for HBI measured as the lowest weekly blood count recorded over 6 weeks after completion of radiation. Pulmonary toxicity assessed by monthly chest X-ray and physical examination up to 6 months.

those that completed at least 3 cycles of chemotherapy (26) was 48·5 weeks. The error in estimating 2-year survival was considered too large because of the few patients, and this is not included in our results. When assessing survival according to stage (Table VI) there was a definite difference in favour of CC in the subgroup of patients with advanced disease, whether or not the patients that did not complete the treatment were included in the analysis. There was no statistically significant difference in the median survival of the *complete responders* group of patients treated with sequential HBI or chemotherapy (51 weeks *vs* 62 weeks). However, there was a statistical difference in favour of CC in the median survival of the *partial responders* (24 weeks for CC *vs* 15 weeks for HBI, $P=0{\cdot}05$, log-rank test).

We were able to switch treatments during progression of the disease in 22 patients; 60% obtained a second remission that lasted a median of 12 weeks. Of 15 patients that relapsed after sequential HBI and were transferred to CC, 8 obtained a second remission that lasted a median of 18 weeks.

Non-fatal diffuse radiation interstitial pneumonitis was seen in 13% of the patients treated with upper HBI in the initial primary treatment arm, with a peak incidence averaging 6 weeks. All 4 cases were examined *post mortem*, and the diagnosis was proven. There was no diffuse pneumonitis in the patients switched to HBI from CC. Marrow toxicity was common with both CC and sequential HBI. We found that the incidence of marrow toxicity increased when the patients that relapsed were switched to the alternative treatment modality (Table II). It was not life-threatening, but mainly severe–moderate toxicity (see Table VI for definition of marrow toxicities). It was most noticeable in the patients receiving sequential HBI as a second treatment after relapsing from the remission induced by CC. All the HBI patients had weekly peripheral-blood cell counts for 5 weeks. The CC patients were followed every 4 weeks and peripheral-blood cell counts were done at that time. The average nadir of white-cell and platelet counts after upper HBI occurred at 21 days, and for the lower HBI at 25 days. Forty-seven per cent of patients on CC needed a delay or modification of chemotherapy due to marrow toxicity.

Extreme anorexia with >20% body-wt loss was seen in 5/34 patients treated with CC and in 8/30 patients treated with HBI, necessitating intraparenteral hyperalimentation in all 5 patients in the CC group and in 4/8 patients in the HBI group. The commonest acute toxicity after upper HBI was severe vomiting, nausea, increase in temperature, tachycardia and hypotension that lasted for 4–6 h ,with complete recovery after 12 h, which was not seen during the lower HBI. There was no instance of deaths related primarily to opportunistic infections, with no proven instances of mycoplasm, pneumocystic carinii or other opportunistic infections producing diffuse interstitial pneumonitis. Five patients had severe Gran-negative septicaemia, necessitating aggressive treatment with antibiotics, all of them while receiving treatments after crossover for recurrence.

Recurrent disease in the form of brain metastasis was observed in 8/30 patients treated with upper HBI as a primary treatment and in 11/34 patients treated with CC, without prophylactic brain irradiation. Necropsy examinations were performed in 20% of expired patients in both treatment arms. All showed failure to control the tumour at the primary irradiated intrathoracic region, as well as the distal metastases.

### DISCUSSION

Although partial or complete tumour responses are currently easily obtained in patients with SCLC treated with systemic chemotherapy with or without local radiation to the primary, the maintenance of this remission for years, achieving eventually the same survival as the normal population corrected for the same age, is a much more diffcult task. This is partly due to tumour resistance to drugs and radiation and partly to the inability to maintain a chemotherapeutic regimen for long because of normal-tissue toxicity, particularly of the marrow.

We chose to investigate the efficacy of wide-field irradiation in this disease in the hope that, if proved efficient, it could eventually be used as a maintenance or consolidating agent. To that end we attempted to assess its therapeutic efficacy (tumour response/toxicity) not only as a single agent but also in a small group of patients that received in their management both CC and HBI (patients that were switched to the alternate modality at relapse).

We decided to compare its efficacy as a single modality in previously untreated patients with advanced or early disease, so that a fair comparison could be made with the same type of patients treated with what was considered at the initiation of the study in 1978, as the best current conventional systemic chemotherapy (Hansen *et al.*, 1977).

The sequential HBI technique produced little inconvenience to the patient, since it was administered in 1 day, and there was no need for hospitalization except with the upper HBI, which necessitated the patient being admitted to hospital for 48 h, because of moderate to severe acute GI and systemic symptoms such as nausea, vomiting, fever, and tachycardia, which usually lasted for 5–6 h. In our experience the patient generally becomes symptom-free and ready to begin normal activity in 24 h. This, in addition to the 13% incidence of interstitial diffuse pneumonitis, could be i nproved in the future by fractionating the total dose of radiation over several days.

As can be seen in Table VI, sequential HBI as the primary treatment has acceptable marrow toxicity, but this more than doubles when HBI is used after CC. This does not preclude its use, but it emphasizes the need for extreme caution when planning to use this modality before or after CC. It is important that a second remission of 3–4 months was obtained in 60% of the patients that relapsed and were switched to either HBI or CC. It appears therefore feasible to use HBI after several courses of systemic chemotherapy, provided there is a minimum interval of 5–6 weeks for marrow recovery.

Using the overall rate and duration of response and median survival as endpoints, we found no evidence to reject the null hypothesis that CC and HBI are equally effective. However, on further analysis, considering subgroups of patients with factors that are known to affect prognosis, such as a stage, type of response, whether they completed treatment or not, we have found that in advanced disease and in patients with partial responses the HBI method alone, without prior chemotherapy, appeared to be less efficient than CC alone. Furthermore, the 13% incidence of non-fatal radiation pneumonitis for the group of patients treated with HBI could make CC preferable. From the practical point of view, HBI is less cumbersome and inconvenient to the patient. Both the CC- and HBI-treated patients have the same quality of survival. The incidence of extreme anorexia with gross weight loss and generalized weakness, necessitating frequent hospital admissions for intraparenteral hyperalimentation, was the same for both groups, as was the incidence of opportunistic infections. The pattern of treatment failure was the same for both treatment arms with all the necropsy material showing evidence of disease at the primary as well as distal metastatic sites, suggesting that the "boosting" dose of radiation to the local primary site was insufficient.

When analysing sequential HBI in combination with CC as a maintenance or consolidating agent, it appears that the patients able to receive both treatment modalities and achieving a second remission live longer than the patients whose treatment was not switched, though the toxicity, particularly to marrow, increased in incidence and severity. This could partially be avoided by proceeding with HBI soon after 3 cycles of induction CC.

From our observations we conclude that although sequential HBI as a primary method of treatment is generally as efficient as CC for small-cell lung cancer, the suboptimal response in patients with advanced disease prompts us to recommend its use as a consolidating or maintenance agent in combination with a more effective chemotherapy regimen. At present, in our centre the treatment for early as well as advanced disease consists of 3 months induction of cyclophosphamide (1000 mg/m$^2$ i.v. on Day 1, Adriamycin (45 mg/m$^2$ i.v.) Day 2 and VP-16 (180 mg/m$^2$ i.v.) Days 1–3, in 21-day cycles with concomitant local chest irradiation to a dose of 50 Gy in 25 fractions in a total time of $5\frac{1}{2}$ weeks, followed by consolidation fractionated HBI at 2·5 Gy per fraction in 4 consecutive fractions for a total of 10 Gy.

The combination of CC and HBI should be considered with extreme care in view of the increased incidence of severe marrow toxicity. Although we have not seen an increased incidence of diffuse interstitial pneumonitis at the time of treatment switch, we recommend extreme caution, particularly when using HBI with chemotherapeutic agents known to produce additive effects in normal tissue of target organs.

The authors acknowledge the help of Dr Roger Amy in reviewing the pathology slides, Dr A. R. Turner for his helpful suggestions, Mr T. Drennan, Mr R. Barnett and Dr J. Scrimger and the Planning Section of the Cross Cancer Institute for the invaluable help on the radiation-treatment planning. Special appreciation for the secretarial help of Miss Lisa Skoreyko.

This work was supported by Grant No. 5230 Alberta Heritage Fund for Medical Research Applied Cancer.

### REFERENCES

ALBERTO, P., BRUNNER, K. W., MARZ, G., OBRECHT, J. P., SONNTAG, R. W. (1976) Treatment of bronchogenic carcinoma with simultaneous or sequential combination chemotherapy, including methotrexate, cyclophosphamide, procarbazine and vincristine. *Cancer*, **38**, 2216.

AISNER, J., WHITACRE, M., VAN ECHO, D. A., ESTERHAY, R. J., JR, WIERNIK, P. H. (1980) Alternating non-cross resistant combination chemotherapy for small cell carcinoma of the lung (SCCL). *Proc. Am. Assoc. Cancer Res. Am. Soc. Clin. Oncol.*, **21**, 453.

COHEN, M. H., FOSSIEK, B. E., IHDE, D. C. & 4 others (1978) Chemotherapy of small cell lung cancer: Results and concepts. In *Progress in Cancer Research and Therapy, 2* (Eds. Muggia & Rozencweig). New York: Raven Press. p. 566.

DANIELS, J. R., CHAK, L., ALEXANDER, M. & 4 others (1980) Oat cell carcinoma: Alternating compared with sequential combination chemotherapy. *Proc. Am. Assoc. Cancer Res. Am. Soc. Clin. Oncol.*, **21**, 346.

FELD, R., PRINGLE, J., EVANS, W. K. & 6 others (1979) Combined modality treatment of small cell carcinoma of the lung (SCCL). *Proc. Am. Assoc. Cancer Res. Am. Soc. Clin. Oncol.*, **20**, 312.

FRYER, C. J. H., FITZPATRICK, P. J., RIDER, W. D. & POON, P. (1978) Radiation pneumonitis: Experience following a single dose of radiation. *Int. J. Radiat. Oncol. Biol. Phys.*, **4**, 936.

GLODE, L. M., HARTMANN, D. & ROBINSON, W. A. (1980) The acute and cumulative toxicity of chemotherapy in small cell carcinoma of the lung with and without bone marrow support. *Proc. Am. Assoc. Cancer Res. Am. Soc. Clin. Oncol.*, **21**, 488.

GRECO, F. A., EINHORN, L. H., RICHARDSON, R. L. & OLDHAM, R. K. (1978) SCLC: Progress and perspectives. *Semin. Oncol.*, **5**, 335.

GREEN, R. A., HUMPHREY, E., CLOSE, H. & PATNO, M. E. (1969) Alkylating agents in bronchogenic carcinoma. *Am. J. Med.*, **46**, 525.

HANSEN, H. H., SELAWRY. O. S., SIMON, R. & 4 others (1976) Combination chemotherapy of advanced lung cancer. *Cancer*, **38**, 2207.

HANSEN, H. H., DOMBERNOWSKY, P., HIRSCH, F. & RYARD, J. (1977) Intensive combination chemotherapy plus localized or estensive radiotherapy in small cell anaplastic bronchogenic carcinoma. *Proc. Am. Assoc. Cancer Res. Am. Soc. Clin. Oncol.*, **21**, 350.

IHDE, D. C., MAKUCH, R. W., COHEN, M. H., BUNN, P. A., MATHEWS, M. J. & MINNA, J. D. (1979) Prognostic implications of sites of metastases in patients with small cell carcinoma of the lung (SCCL) given intensive chemotherapy. *Proc. Am. Assoc. Cancer Res. Am. Soc. Clin. Oncol.*, **20**, 264.

LIVINGSTON, R. & MIRA, J. (1980) Non-cross resistant combinations in patients (PTS) with extensive small cell lung cancer. *Proc. Am. Assoc. Cancer Res. Am. Soc. Clin. Oncol.*, **21**, 449.

OLDHAM, R. K. & GRECO, F. A. (1980) Small cell lung cancer: A curable disease. *Cancer Chemother. Pharmacol.*, **4**, 177.

PENDERGRASS, K. V., ABELOFF, M. D., ETTIGER, D. S., BURKE, P. T., ORDER, S. E. & KOURI, N. (1980) Intensive timed sequential combination chemotherapy and adjunctive radiotherapy in extensive stage small cell carcinoma of the lung. *Proc. Am. Assoc. Cancer Res. Am. Soc. Clin. Oncol.*, **21**, 447.

SALAZAR, O. M. & CREECH, R. H. (1980) The state of the art toward defining the role of radiation therapy in the management of small cell bronchogenic carcinoma. *Int. J. Radiat. Oncol. Biol. Phys.*, **6**, 1103.

SALAZAR, O. M., CREECH, R. H., RUBIN, P. & 5 others (1980) Half body and local chest irradiation as consolidation following response to standard induction chemotherapy for disseminated SCLC. *Int. J. Radiat. Oncol. Biol. Phys.*, **6**, 1093.

SALAZAR, O. M., RUBIN, P., BROWN, J. C., FELDSTEIN, M. L. & KELLER, B. E. (1976) Predictors of radiation response in lung cancer: A clinico–pathological analysis. *Cancer*, **37**, 2650.

SALAZAR, O. M., RUBIN, P., KELLER, B. & SCARANTINO, C. (1978) Systemic (half body) radiation therapy: Response and toxicity. *Int. J. Radiat. Oncol. Biol. Phys.*, **4**, 937.

URTASUN, R. C., BELCH, A. R., HIGGINS, E. M., SAUNDERS, W. M. & MCKINNON, S. (1980) Whole body irradiation of small cell carcinoma of the lung (SCLC) compared to three drug chemotherapy. *Proc. Am. Assoc. Cancer Res. Am. Soc. Clin. Oncol.*, **21**, 451.

VAN, DYK, J., KEANE, T. J., KAU, S. & RIDER, W. D. (1981) Radiation pneumonitis following large single dose irradiation: A re-evaluation based on absolute dose to lung. *Int. J. Radiat. Oncol. Biol. Phys.*, **7**, 467.

VINCENT, R. G., WILSON, H., LANE, W., RAZA, S. & CHEN, T. (1980) Sequential chemotherapy of small cell carcinoma of the lung. *Proc. Am. Assoc. Cancer Res. Am. Soc. Clin. Oncol.*, **21**, 453.

WOODS, R. L., TATTERSALL, M. H. N. & FOX, R. M. (1981) Hemi-body irradiation (HBI) in "poor prognosis" small cell lung cancer (SCLC) *Proc. Am. Assoc. Cancer Res. Am. Soc. Clin. Oncol.*, **22**, 502.